# Gating Mechanism of the Voltage-Gated Proton Channel Studied by Molecular Dynamics Simulations

**DOI:** 10.3390/molecules27072277

**Published:** 2022-03-31

**Authors:** Thi Tuong Vy Phan, Myunggi Yi

**Affiliations:** 1 Center for Advanced Chemistry, Institute of Research and Development, Duy Tan University, 03 Quang Trung, Hai Chau, Danang 550000, Vietnam; phanttuongvy4@duytan.edu.vn; 2 Faculty of Environmental and Chemical Engineering, Duy Tan University, 03 Quang Trung, Hai Chau, Danang 550000, Vietnam; 3 Department of Biomedical Engineering, Pukyong National University, Busan 48513, Korea; 4 Industry 4.0 Convergence Bionics Engineering, Pukyong National University, Busan 48513, Korea

**Keywords:** gating mechanism, Hv1 proton channel, water-wire, molecular dynamics simulations

## Abstract

The voltage-gated proton channel Hv1 has important roles in proton extrusion, pH homeostasis, sperm motility, and cancer progression. The Hv1 channel has also been found to be highly expressed in cell lines and tissue samples from patients with breast cancer. A high-resolution closed-state structure has been reported for the mouse Hv1 chimera channel (mHv1cc), solved by X-ray crystallography, but the open-state structure of Hv1 has not been solved. Since Hv1 is a promising drug target, various groups have proposed open conformations by molecular modeling and simulation studies. However, the gating mechanism and the open-state conformation under the membrane potential are still debate. Here, we present a molecular dynamics study considering membrane potential and pH conditions. The closed-state structure of mHv1cc was used to run molecular dynamics (MD) simulations with respect to electric field and pH conditions in order to investigate the mechanism of proton transfer. We observed a continuous hydrogen bond chain of water molecules called a water-wire to be formed through the channel pore in the channel opening, triggered by downward displacement of the S2 helix and upward movement of the S4 helix relative to other helices. Due to the movement of the S2 and S4 helices, the internal salt bridge network was rearranged, and the hydrophobic gating layers were destroyed. In line with previous experimental and simulation observations, our simulation results led us to propose a new gating mechanism for the Hv1 proton channel, and may provide valuable information for novel drug discovery.

## 1. Introduction

The voltage-gated proton channel, Hv1, plays an important role in the pH control of the cytoplasm and internal organelles [1]. The Hv1 channel is expressed in many species and is involved in a range of specialized physiological processes, such as regulation of membrane potential and pH in human cardiac fibroblasts [2], bioluminescence in dinoflagellates [3], acid extrusion in snail neurons during an action potential [4], fertilization in amphibians and human sperm [5], acid secretion in airways [6], and the facilitation of B-cell activation during an immune response [7]. The primary characterized function of the proton channels is electron balance mediated by the H^+^ flux during phagocytic respiratory burst. The Hv1 channel is regarded as the key for the optimization of NADPH oxidase activity with decreasing pH in the phagosomal lumen and cytoplasm [8]. The Hv1 plays an important role in altering the function of this channel to entail several physiological dysfunctions. For instance, in human breast cancer cells, the aberrant expression of the Hv1 channels was shown to correlate with the propensity for acquiring metastatic features [9]. Hv1 activity has been associated with invasive and metastatic phenotypes of different tumors (e.g., breast and colorectal cancer) [9]. Due to the relevancy of the Hv1 in physiology and pathology, it has become a focus of active research as a potential pharmacological target. As a voltage-sensing domain able to conduct protons, one of the most intensively studied questions concerning the biophysical properties of voltage-gated proton channels is related to the mechanism by which voltage-dependent gating takes place.

**Figure 1:**
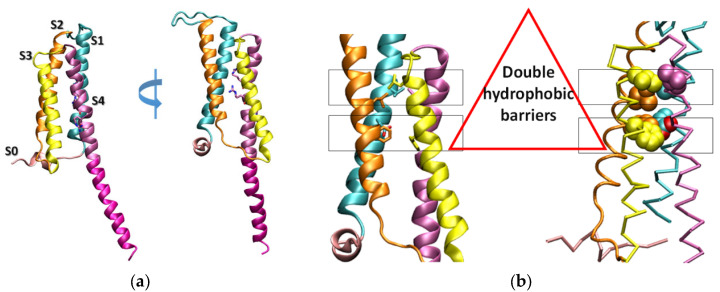
(**a**) The crystal structure of mHv1cc. The ribbon model of the overall crystal structure of mHv1cc, viewed from two different angles rotated 90° along the membrane bilayers. The four transmembrane segments, or helices S1 (cyan), S2 (orange), S3 (yellow), and S4 (purple), connected to the cytoplasmic coiled-coil region (magenta) are shown. The N-terminal cytoplasmic helix (S0) is colored in pink. Three arginine residues, Arg201, Arg204, and Arg207, in S4 are shown as sticks. (**b**) The double hydrophobic layers of the mHv1cc. The residues in two layers are shown by sticks (left) and spheres in the 90° rotated one (right). The upper layer close to the extracellular side consists of four highly conserved hydrophobic residues (Val112 (S1), Leu143 (S2), Leu185 (S3), and Leu197 (S4)). The lower layer consists of two hydrophobic residues—Phe146 (S2) and Phe178 (S3)—and one hydrophilic residue—Asp108 (S1, oxygen atom colored in red).

Classical channels such as voltage-gated sodium (Nav), potassium (Kv), and calcium (Cav) channels all share a similar architecture: first, four transmembrane segments (S1–S4) form the voltage-sensor domain (VSD); second, two transmembrane segments (S5–S6) form the pore domain [10]. The Hv1 has a VSD that is similar to the VSD of the canonical voltage-gated ion channel, but lacking a pore domain; thus, the proton permeates through the VSD [11]. The Hv1 consists of two functional domains: the VSD and the cytoplasmic coiled-coil domain. The crystal structure of mouse Hv1 chimera construct (mHv1cc) shows that the longest fourth transmembrane helix (S4) is directly connected to the cytoplasmic coiled-coil region, forming a long, slightly bent helical structure [12] (Figure 1a). The mHv1cc crystal structure has two hydrophobic layers, and the Asp108, which is located in the hydrophobic layers, is critical for selective proton permeation (Figure 1b). Two hydrophobic layers work as a shield to prevent the permeation of water molecules.

Proton current was identified in snail neurons in the 1980s, and the genes encoding for human and murine Hv1 were discovered in 2006 [4,11]. In 2014, the structure of mHv1cc was putatively solved by X-ray crystallography in a closed state [12]. In all of the previous studies, the homology modeling of Hv1 was built based on the structures of Kv, Nav, and the Kv1.2–Kv2.1 paddle chimera as templates, with sequence identities of less than 30% [13,14,15]. Recently, voltage-dependent structural models and the inhibitor (2GBI) binding of the human Hv1 channel were reported using the homology modeling of Hv1 based on the mHv1cc crystal structure to run molecular dynamics (MD) simulations [16,17,18]. Despite the previous research attempts, the understanding of the gating mechanisms of Hv1 is still in debate, especially with the gating mechanism under the membrane potential.

In this study, the X-ray structure of the mHv1cc was used to perform MD simulations while considering the electric field and the pH conditions, in order to investigate the gating mechanism of mHv1cc. Since all of the available structural models and molecular dynamics studies modeled human Hv1 by means of homology modeling, which may introduce artifacts, we decided to use the original sequence and structure used in the X-ray structure. In addition, mHv1cc is a voltage-sensitive functional proton channel [12], and Lee, M. et al. used the structure of mHv1cc structure for molecular dynamics simulations to investigate the closed state [15]. After modeling the missing loop regions in the X-ray structure and assigning protonation states according to pH conditions, we built a monomeric channel and imbedded it into explicitly solvated and pre-equilibrated lipid bilayers to run molecular dynamics simulations with various membrane potentials (electric fields, see Materials and Methods).

## 2. Results

### 2.1. Water-Wire in the mHv1cc Channel

**Figure 2:**
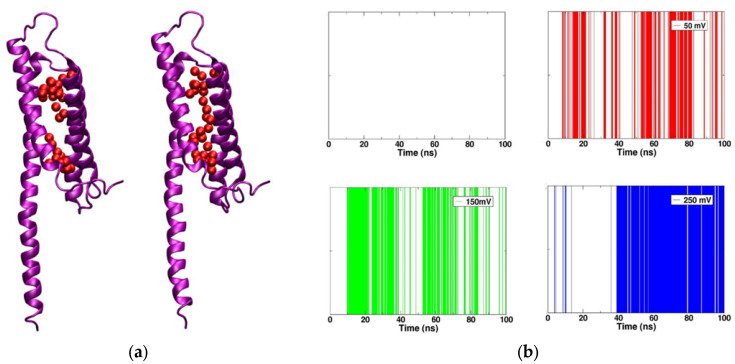
(**a**) Discontinuous water-wire (left) and continuous water-wire (right). The four transmembrane segments of mHv1cc are represented by purple ribbons; oxygen atoms of water molecules are shown as red spheres. (**b**) The water-wire formation in the channel pore during the simulations. The white-colored stripe, or background, represents a discontinuous water-wire, and the red, green, and blue stripes represent a continuous water-wire.

First, we focused on analyzing the formation of the continuous water-wire under the various electric fields in simulations 2 (50 mV), 3 (150 mV) and 4 (250 mV). Discontinuous water-wire was observed in the mHv1cc throughout the full trajectory of simulation 1 (0 mV) (Figure 2a). Meanwhile, a continuous water-wire was exhibited in the channel in the other simulations with the membrane potentials (Figure 2a). Among the three simulations with various voltages, simulation 4 (250 mV) generated the most frequent and long-lived water-wire during the 100 ns trajectory. When the membrane potential was increased from 50 mV to 150 mV and 250 mV, the proportion of continuous water-wire increased from 19.53% to 28.71% and 48.84%, respectively, indicating the successful construction of a model in a voltage-gating manner (Figure 2b).

### 2.2. The Effect of the Electric Field on the Channel

**Figure 3:**
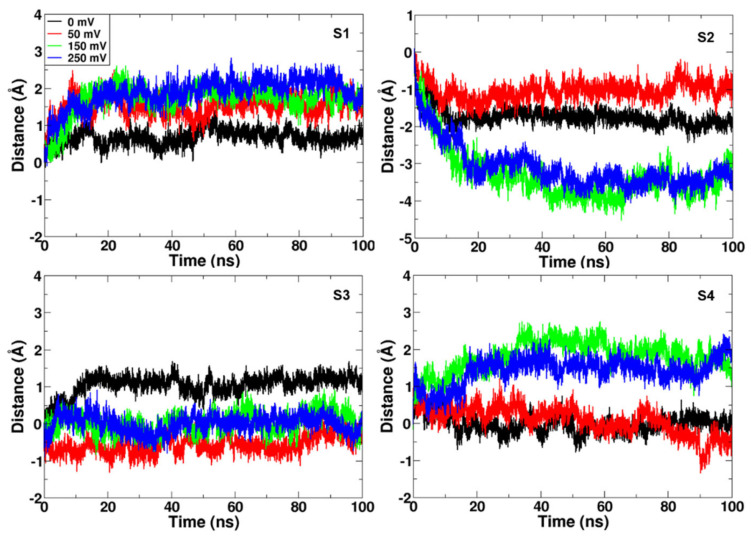
The moving distances of the four helices along the normal to the membrane bilayers (*z*-axis) relative to the center of the four helices. Alpha carbon atoms of each helix were used to calculate the distance as a reference. The analyses for helices S1 to S4 are plotted separately and colored according to the four simulations; black, red, green, and blue lines for 0 mV, 50 mV, 150 mV and 250 mV, respectively. Significant downward displacement of the S2 and upward movement of the S4 helices are shown in the 150 mV and 250 mV simulations relative to the 0 mV and 50 mV simulations.

To understand the effect of the membrane potential on the channel, we calculated the overall movement of each helix along the *z*-axis (normal to the membrane plane) relative to the center of the whole channel (except the coiled-coil region) as a reference (Figure 3). The results indicated that the S3 helix was quite stable in all simulations, and the S2 and S4 helices moved significantly in the simulations with applied electric field, while these helices were relatively stable in the simulations without electric field. Root mean squared deviation (RMSD) calculations also indicated greater deviations from the initial conformation in simulation 3 (150 mV) and simulation 4 (250 mV) (Figure S1). At first glance, we can guess that the opposite movements of the S2 helix and the S4 helix may be related to the gating mechanism of the Hv1 (Figure 3).

#### 2.2.1. The Upward Movement of the S4 Helix

We calculated the center of each helix, as illustrated in Figure 3. The upward motion of the S4 helix under higher electric fields is shown. Significant movement of the S4 helix was observed in simulation 3 (150 mV) and simulation 4 (250 mV). Meanwhile, simulation 1 (0 mV) and simulation 2 (50 mV) showed smaller movements of the S4 helix. The upward motion of a segment is considered to be a common mechanism of sensing in the voltage-gated channel family [19]. Using cysteine accessibility measurements and a voltage-clamp fluorimeter, Gonzalez et al. pointed out that the S4 helix moved upward (outward to the extracellular side) during channel opening [20].

#### 2.2.2. The Movement of Sidechains of Arg Residues on S4 Helix

**Figure 4:**
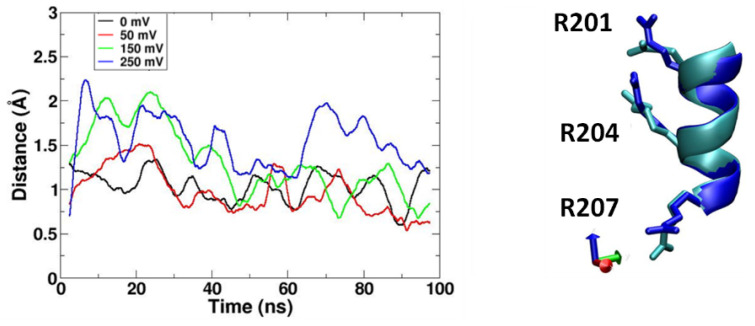
(**Left**) The moving distance of the side chains of three arginine residues on the S4 helix along the normal to the membrane bilayers. (**Right**) Superposition of the S4 helix (ribbon representation with the backbone of residues 201 to 207) between a closed conformation (cyan, simulation with 0 mV) and an open conformation from simulation 4 (blue, 250 mV).

Three highly conserved arginine residues are located at every third position from Arg201 to Arg207 along the S4 helix (Figure 1a and Figure 4) in voltage-gated proton channels, and the membrane potential is sensed by voltage-sensitive phosphatases [19]. This plays an important role in driving the conformational changes that are associated with channel gating and enzyme activation [19]. The electric field can lead not only to the movement of the S4 helix, but also to the movement of the sidechains of Arg on the S4 helix (Figure 4). Actually, the upward movement of these basic amino acids under the membrane potential is the driving force for the upward movement of the S4 helix. These movements can be observed clearly in simulation 4 (250 mV). Simulation 4 (250 mV) showed the most frequent formation of continuous water-wire, which was about two times higher than that of simulation 2 (50 mV) (Figure 2b). The upward motion of the S4 helix and the basic residues may induce some changes of their salt-bridge partners.

#### 2.2.3. The Change of the Salt-Bridge Network

**Figure 5:**
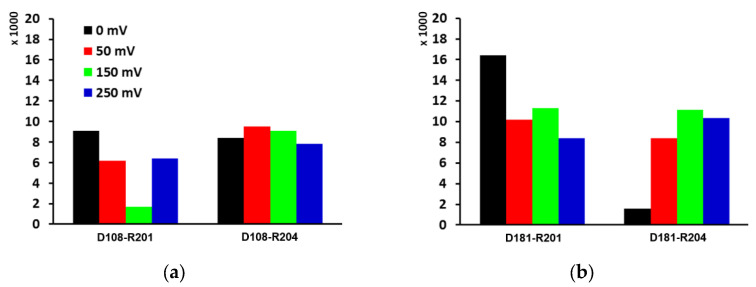
The number of hydrogen bonds of (**a**) Asp108 (on S1 helix) formed with Arg201 (S4) and Arg204 (S4), and of (**b**) Asp181 (S3) formed with Arg201 (S4) and Arg204 (S4) over the simulation times of four simulations. Upon voltage activation, Arg204, the lower residue on the S4 helix, forms a salt-bridge frequently with Asp108 on the S1 helix and Asp181 on the S3 helix. Hydrogen bonds are calculated with a cut-off distance of 3.4 Å and an angle of 30 degrees.

The interactions between helices are important for the integrity of channel formation, especially for small membrane proteins [21]. In Hv1, the salt-bridge network plays a critical role in the stability of the channel [22]. Changes in the salt-bridge network are one of the conditions required for the opening of the channel. In the closed state, Asp108 on the S1 helix forms a salt-bridge with both Arg201 and Arg204 on the helix S4. When the S4 helix moved up, the formation of the Arg201-Asp108 salt-bridge became less frequent (Figure 5a). Throughout the trajectory of simulation 1 (0 mV), Asp181 on the S3 helix mostly formed a salt-bridge with Arg201 on the S4 helix. However, when the S4 moved up under the electric field, Asp181 switched to both Arg201 and Arg204 as salt-bridge partners (Figure 5b). This indicates that the Asp108 on S1 and the Asp181 on S3 switch their salt-bridge partners from the upper residue (Arg201) to the lower residue (Arg204) under the electric field. Additionally, Arg201 switched its hydrogen bond partner from Asp108 to Asp181, and Arg204, too, switched its salt-bridge partner from Asp108 to Asp181 (Figure 5).

#### 2.2.4. The Downward Movement of the S2 Helix Relative to Other Helices

So far, no literature has mentioned the downward displacement of the S2 helix when the channel opens. Our electric field simulations show two significant movements. One is the upward movement of the S4 helix, and the other is the downward movement of the S2 helix (Figure 3). Especially in simulation 3 (150 mV) and simulation 4 (250 mV), significant downward movement (−3~−4 Å) along the *z*-axis of the S2 helix was observed (Figure 3). Since the distances along the *z*-axis were measured with respect to the center of the four helices, the relative downward displacement of the S2 helix was expected due to the upward movement of the other helices. The applied electric field moved three Arginine residues, resulting in upward movement of the S4 helix, and then the neighboring helices S1 and S3 moved up along with the S4 helix due to the strong interactions including the salt-bridges (Figure 5). However, the S2 helix is located on the opposite side to the S4 helix, across the channel pore, and has fewer interactions with the S4 helix. The S2 helix is electrically neutral and is the least hydrophilic among the helices. The isoelectric points of the four helices have been estimated to be 5.41, 7.20, 3.07 and 12.5 for the S1 to S4 (Phe191~Met217 only for transmembrane part of the S4) helices, respectively (GPMAW lite) [23]. Even though S4 interacts with S3 through the salt-bridge, the most negatively charged helix, S3, moved up the least under the electric field (Figure 3). Therefore, the S2 helix stably remaining neutral in the membrane bilayers can be interpreted as downward movement relative to the other helices.

This downward displacement of the S2 helix, however, appears to be highly correlated with the channel opening. Similar to the upward movement of the S4 helix, the downward movement of the S2 helix was also rather consistent with the frequency of the continuous water-wire formation (Figure 2b). A comparison of the amplitudes between the movements of S2 and S4 helices along the *z*-axis suggests that the downward displacement of the S2 helix is also the main factor contributing to the opening process of the channel. Therefore, we further investigated the S2 motion and channel gating.

### 2.3. Opening of the Gate

#### 2.3.1. Downward Movement of Phe146

**Figure 6:**
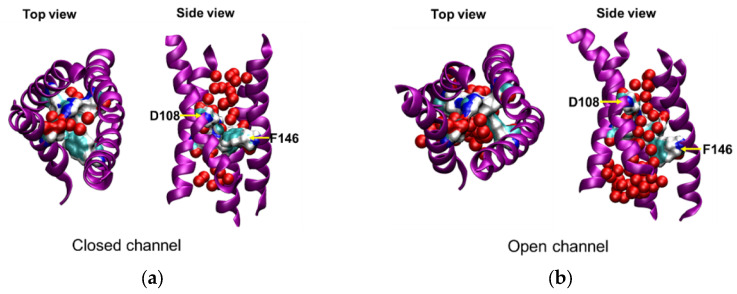

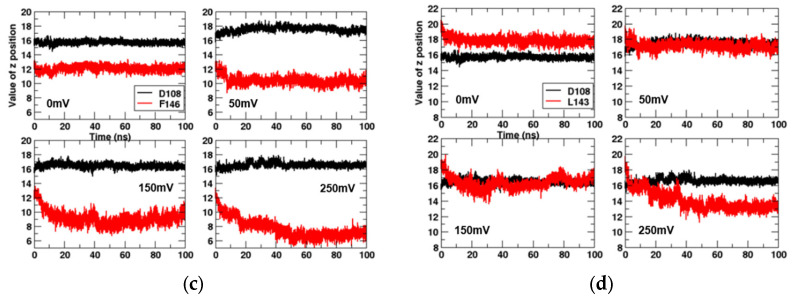
(**a**,**b**) Snapshots of a discontinuous water-wire and a continuous water-wire through the closed and open channel pores, respectively. The transmembrane part of the channel is represented by the purple ribbons, and the four residues of the gate are represented by the surface style (cyan: carbon; blue: nitrogen; and white: hydrogen). The oxygen atoms of water molecules in the channel pore are represented by red spheres. (**c**) The centers of sidechain of Asp108 and Phe146, and (**d**) Asp108 and Leu143 along the *z*-axis are shown. The Z position was calculated after superimposing the channel using Asp108 residue as the reference.

To explain the reason for the upward movement of the S4 helix and the downward movement the S2 helix leading to the opening of the channel, the rearrangement of the residues forming the gate was analyzed. The most constricted point of the channel pore was formed by four residues: Asp108 (S1), Phe146 (S2), Phe178 (S3), and Arg204 (S4) (Figure 1b and Figure 6a). The positions of Phe146 along the *z*-axis were calculated and compared with Asp108, and it was observed that Phe146 moved away from this gate when the S2 helix moved down under the electric field (Figure 6b,c). Instead, Leu143, another hydrophobic amino acid and an upper residue on the S2 helix, replaced it and contributed to this gate (Figure 6d). Due to the lower hydrophobicity and smaller sidechain of Leucine compared to Phenylalanine, the gate became weaker. In addition, the upward movement of Arg204 (S4) also cause the gate to weaker further. Thus, a continuous water-wire was formed. As shown in Figure 6b, a continuous water-wire was formed through the space between Asp108 and Phe146.

#### 2.3.2. Pore Radius of the Channel

**Figure 7:**
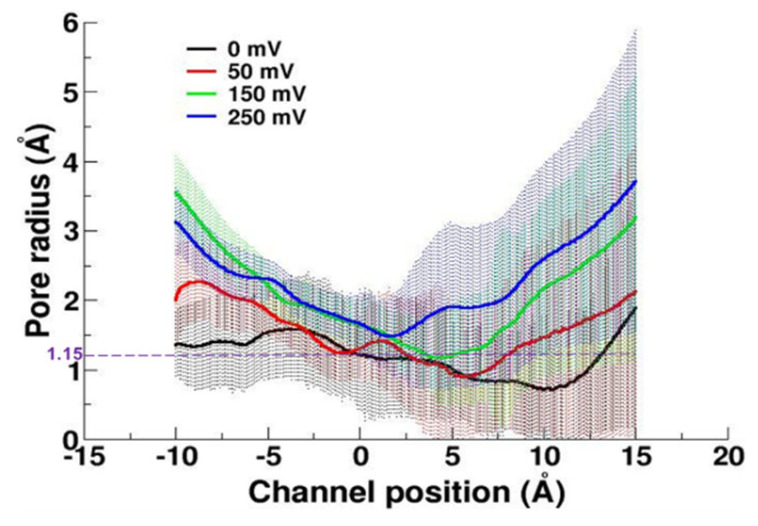
Radii of the channel pore of four simulations.

A radius of 1.15 Å in a channel pore is considered to be the minimum requirement for water molecules to pass through [24]. Based on the pore radius calculations, it can be seen that the radius at the most constricted point consistently increased with the increase in the electric field (Figure 7). The pore radius analysis indicates that the hydrophobic layer 1 (around 10 Å on the channel position along the pore) did not work as a gate for water molecules under the electric field anymore. In a previous study, a similar downward displacement of the most constricted pore region was observed, too [16].

## 3. Discussion

Since Hv1 is considered to be a promising drug target, most (if not all) molecular modeling studies for channel activation, drug binding, and open conformations have been modeled for human Hv1. We were not able to find any molecular modeling or MD simulation studies using mHv1cc itself, which is the only functional proton channel of Hv1 analog possessing a high-resolution X-ray structure [12]. In this study, four MD simulations were conducted considering membrane potential (electric field) and pH conditions in order to obtain the “open state” channel conformation from the “closed state” of the mHv1cc X-ray crystal structure. We were able to model a functional channel in a voltage-dependent manner, which is consistent with experimental observations [12].

Unlike most of the previous studies [16,17,25,26,27], some groups have proposed and investigated proton transfer through the protonation and deprotonation of Asp108 (Asp112 in human Hv1) using the empirical valance bond (EVB) method and reduced quantum simulations [15,28]. In this study, the mHv1cc exhibited a continuous water-wire when the channel was activated by membrane potential (Figure 2). In cell expression systems, the proton current of the full-length M2 channel is very low, at 210 protons/s at pH 6.2 [29]. The proton transfer in the M2 channel takes place via the protonation and deprotonation of amino acid in the pore [30]. Cherny V. V. et al. reported the proton current of the Hv1 channel to be 2.4 × 10^4^ protons/s at pH 6.5 and 9 × 10^4^ protons/s at pH 5.5, values which are much higher than the current of the M2 channel [31]. Thus, the protonation and deprotonation process may not be suitable to explain the proton transfer rate of Hv1. Therefore, protons are putatively transferred through the water-wire by “Grotthuss hopping” under the membrane potential and gradient of proton concentration (pH difference) [32].

**Figure 8:**
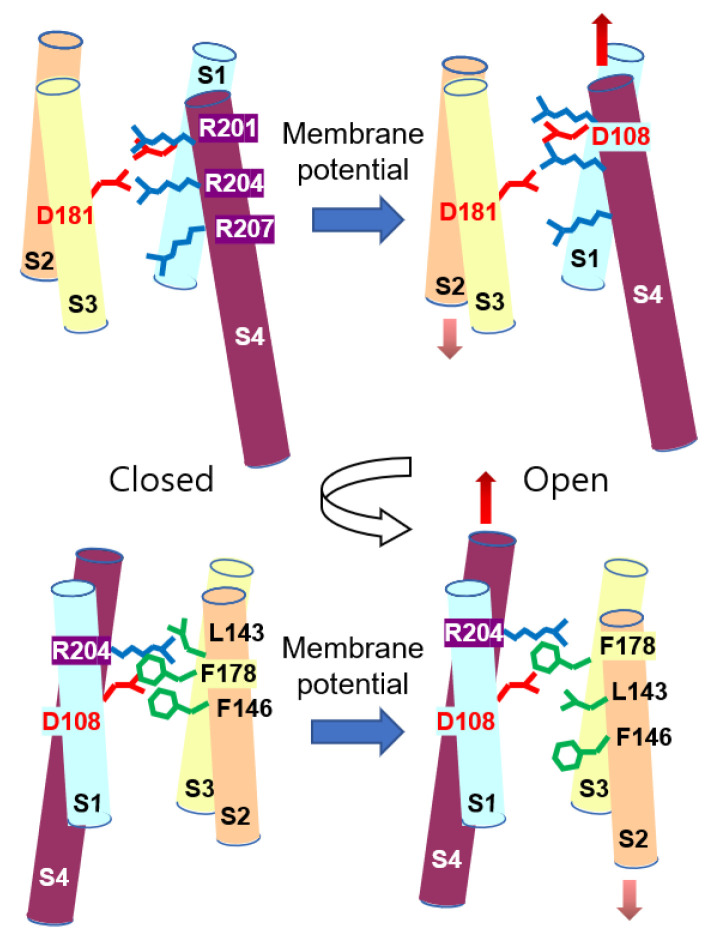
Proposed gating mechanism of Hv1 voltage-gated proton channel.

We observed the upward displacement of the S4 helix to have a strong positive net charge in channel activation, and this is consistent with previous studies [14,15,16,17,18,20]. Along with the upward movement of the S4 helix, a change in the hydrogen bond network (Figure 5) was observed, which is consistent with previous studies [14,16,17]. However, it was a surprise to us that no study has reported the downward displacement of the S2 helix. Due to the strong inter-helical interactions including the salt-bridges, the neighboring S1 and S3 helices moved up along with the S4 helix, resulting in the downward displacement of the S2 helix relative to the other helices. Along with the S2 helix, Phe146, which is essential for channel gating, moved down to open the channel (Figure 6). A similar downward displacement of Phe146 on the S2 helix was observed in a previous study [16], and Phe146 is critical for channel gating, as shown in electrophysiological studies [33,34]. The conformational changes, including backbone and side chains and the rearrangement of the hydrogen bond network, switched the channel from a closed state to an open state under the membrane potential (Figure 8).

In conclusion, the S4 helix, together with the S1 and S3 helices, moves up under the membrane potential, resulting in the relative downward displacement of the S2 helix. Therefore, Phe146 (Phe150 in human Hv1), the key residue for the gating, moves down on the S2 helix to open the channel, producing a continuous water-wire. This study improves the understanding of the gating mechanisms of Hv1 under the membrane potential and provides the open state structure upon the conformational changes that occur throughout the gating. Knowledge-based drug design is becoming a fast and cost-efficient tool. Structure-based drug discovery is a well-known example. Virtual screening by molecular docking requires the structure of a drug target, and recent computational advances use artificial intelligence and machine learning with genomic, proteomic, and structural information as input data. Therefore, this study may provide valuable information for novel drug discovery.

## 4. Materials and Methods

The closed state of mHv1cc (PDB ID: 3WKV) was used to set up the initial conformation of all MD simulations [12]. The missing loops in the X-ray crystal structure were modeled using the SWISS-MODEL [35]. To obtain the stable conformation, one MD simulation with a complete mHv1cc channel was run without the electric field and under neutral pH conditions. Two histidines that coordinate with the zinc ion were assigned: Hsd136 (electrically neutral histidine protonated at the delta nitrogen) and Hse189 (neutral histidine protonated at the epsilon nitrogen). Then, the protein was embedded in a fully hydrated and pre-equilibrated 1,2-dioleoyl-sn-glycero-3-phosphocholine (DOPC) bilayer.

Using the stable closed conformation from the above simulation, three simulations with different membrane potentials (50, 150, 250 mV) were set up to mimic the experimental conditions [12]. The applied electric field is perpendicular to the membrane plane, or along the *z*-axis, and has a magnitude E = V/z (mV/Å), in which V is the membrane potential and z is the thickness of the membrane (Å). The thickness of the membrane bilayer was approximated as 36 Å based on the average over the MD simulations. The simulations were named simulation 1 (0 mV), simulation 2 (50 mV), simulation 3 (150 mV), and simulation 4 (250 mV). To mimic the pH conditions of the extracellular pH (pH_out_) and intracellular pH (pH_in_) of the experimental condition [12], the histidines on the extracellular side were deprotonated (electrically neutral), and the intracellular side histidines were protonated (positive, both delta and epsilon nitrogens were protonated). The CHARMM force field was used for all simulations [36]. These simulation systems were neutralized and solvated with KCl (0.15 M) and 19,997 explicit TIP3P water molecules.

All simulations were performed by NAMD with CHARMM 36 force field under the periodic boundary conditions [36,37]. Initially, 1000 steps of energy minimization were performed to remove bad contacts. Then, the MD simulations were initiated by gradually heating the systems from 10 to 298 K under the constant-volume condition for 60 ps. The simulation systems were switched to conditions with constant pressure and temperature afterwards. The simulation systems were equilibrated in the course of 200 ps. All heavy atoms in these steps of energy minimization, heating, and equilibration were restrained with a harmonic force constant of 1 kcal/mol/Å^2^. Finally, the harmonic restraints were removed, and the simulations were continued, allowing all atoms in the systems to relax. All bonds involving hydrogen atoms were constrained, allowing an integration time step of 2 fs, and average pressure and temperature were maintained at 1 bar and 298 K. The nonbonded interactions were smoothly truncated from a 10 Å to a 12 Å cutoff, and the particle-mesh Ewald method was used to treat long-range electrostatic interactions [38]. The total length of the MD trajectory of each was 100 ns. All conformational changes were visually inspected and analyzed using the Visual Molecular Dynamics (VMD) program [39]. If simulation times are not shown, all the results were analyzed after the equilibration stage of the simulations. The equilibration stage was determined by RMSD (Figure S1).

We defined the continuous water-wire as water molecules in the channel connecting the extracellular side and the intracellular side when the distances between the two oxygen atoms of all adjacent water molecules were less than 3.4 Å. Water-wire formation along each simulation trajectory was analyzed as a binary process: ‘yes’ for continuous and ‘no’ for discontinuous. The pore sizes were calculated using all atoms in residue 97 to 120 of helix 1, 133 to 157 of helix 2, 166 to 188 of helix 3, and 193 to 214 of helix 4 using the HOLE program [24].

## Data Availability

Not applicable.

## Supplementary Materials

The following supporting information can be downloaded at: https://www.mdpi.com/article/10.3390/molecules27072277/s1, Figure S1: RMSD.
