# Supplementary material for: Gating Mechanism of the Voltage-Gated Proton Channel Studied by Molecular Dynamics Simulations
Source: Molecules. 2022 Mar 31;27(7):2277. doi: 10.3390/molecules27072277 (PMC9000549; doi:10.3390/molecules27072277)
Supplement: Supplementary file 1 [file molecules-27-02277-s001.zip › molecules-1628625-supplementary.pdf]

molecules

Gating mechanism of the voltage-gated proton channel studied by molecular dynamics simulations

Thi Tuong Vy Phan [1,2] and Myunggi Yi [3,4,*]

1 Center for Advanced Chemistry, Institute of Research and Development, Duy Tan University, 03 Quang Trung, Hai Chau, Danang 550000, Vietnam; phanttuongvy4@duytan.edu.vn
2 Faculty of Environmental and Chemical Engineering, Duy Tan University, 03 Quang Trung, Hai Chau, Danang 550000, Vietnam
3 Department of Biomedical Engineering, Pukyong National University, Busan 48513, Korea
4 Industry 4.0 Convergence Bionics Engineering, Pukyong National University, Busan 48513, Korea
* Correspondence: myunggi@pknu.ac.kr; Tel.: +82-51-629-5773

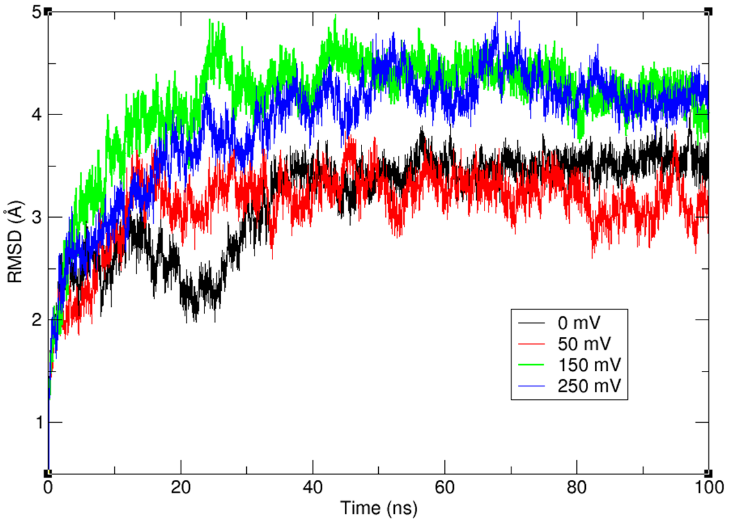

Figure S1. The RMSD of four helices (S1~S4, alpha carbon atoms of residue 95~214 are selected for RMSD calculations).
